# Editorial: Advances in Phage Therapy: Present Challenges and Future Perspectives

**DOI:** 10.3389/fmicb.2021.701898

**Published:** 2021-06-04

**Authors:** Petar Knezevic, Naomi Sulinger Hoyle, Shigenobu Matsuzaki, Andrzej Gorski

**Affiliations:** ^1^Department of Biology and Ecology, Faculty of Science, University of Novi Sad, Novi Sad, Serbia; ^2^Eliava Phage Therapy Center (EPTC), Tbilisi, Georgia; ^3^Medical School, Kochi University, Kochi, Japan; ^4^Hirszfeld Institute of Immunology and Experimental Therapy, Polish Academy of Sciences, Wrocław, Poland

**Keywords:** phage therapy, infection, synergy, phage cocktail, biofilm, endolysin, phage-bacteria interactions, personalized therapy

The emerging multiple- and pan-drug resistant bacterial strains enforce research in the field of new antimicrobial agents and therapeutic strategies, in the combat against life-threatening infections. In the post antibiotic era, bacteriophages have been considered as one of the major solutions to overcome the current medical crisis, offering many advantages over conventional antimicrobials. During the last two decades, a significant progress in phage therapy (PT) is evident, but we still need to identify and fill gaps in the knowledge, complement experience, provide additional proofs of PT efficacy and safety, as well as to reconsider methods and practical approaches to bring beneficial results for human health and well-being. The Research Topic *Advances in Phage Therapy: Present Challenges and Future Perspectives* encompasses the analysis of past experiences of phage application, current state of PT in contemporary medical practice, new original data and consideration of future perspectives (Figure 1).

**Figure 1:**
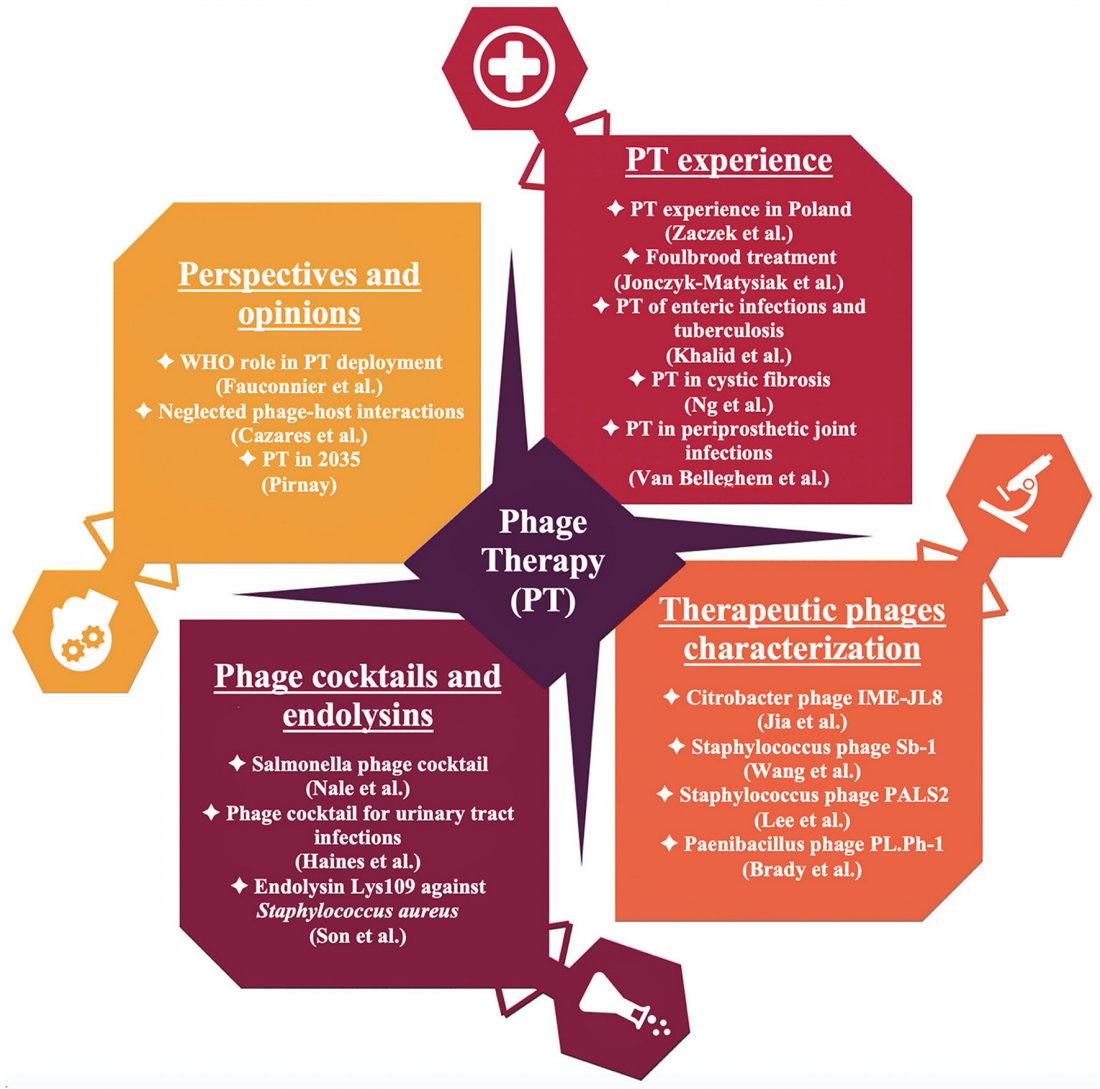
Contribution of the issue *Advances in Phage Therapy: Present Challenges and Future Perspectives* to the state of PT.

## Experience in PT

Since bacteriophage discovery, more than 100 years ago, there have been intentions to introduce phages into therapy for diseases for which bacteria have been etiological agents. When antibiotics were discovered, the aspirations of phage application in therapy were abandoned. However, in several countries, including Poland and Georgia, PT continued to be part of medical practice, to a lesser or greater extent. Today, beside a few completed and ongoing clinical trials, there are several phage therapy units throughout the world (e.g., USA, Belgium, Georgia, Poland, Australia), which continually provide valuable information. In the manuscript by Zaczek et al. the history of phage therapy in Poland was described in details, from the interwar period to the modern days and the establishment of the first Phage Therapy Unit at the Hirszfeld Institute of Immunology and Experimental Therapy in Wrocław in 2005. The special value of this review paper is that it familiarizes us with numerous Polish scientists who have worked in this field but remained insufficiently known to the scientific community.

Khalid et al. reviewed phage application in bacterial foodborne diseases control (typhoid, cholera, shigellosis, *E. coli* caused diarrhea) and tuberculosis, indicating that this approach can be a solution for disease control in developing countries.

In the article presented by Ng et al. phage application in the treatment of *Pseudomonas aeruginosa* infections in patients suffering from cystic fibrosis was reviewed, considering *in vitro* models, cell culture models, and future prospects. Another phage therapeutic application was considered in the review by Van Belleghem et al. who scrutinized major aspects of the prosthetic joint infection (JPI) pathogenesis and biofilm formation, phage properties and their interaction with immune system, their use as supportive therapy in JPI, as well as in prophylaxis.

Besides phage application as alternative remedy for bacterial diseases of humans, they have been considered in the context of beekeeping. Namely, American foulbrood, a disease caused by *Paenibacillus larvae* that infects honeybees (*Apis melifera*) can also be treated by bacteriophages. In the article by Jończyk-Matysiak et al., beside pathogenesis, epidemiology, diagnostics, conventional treatments and antibiotic resistance, particular attention has been paid to phenotypic and genotypic characteristics of *P. larvae* bacteriophages, their exploitation in PT, endolysin production, as well as the limitations of such approach to disease control.

## Therapeutic Phages Characterization

Within the framework of the scientific topic, several new phages were described. A new phage IME-JL8 (family *Siphoviridae*) of fish pathogen *Citrobacter freundii* was described in detail, including *in vitro* lytic efficacy and effect on biofilm. *In vivo* experiments demonstrated promising application of this phage for the prevention and treatment of *C. freundii* fish infections (Jia et al.). A novel jumbo phage PALS2 (family *Myoviridae*) isolated from bird feces was characterized in detail and its anti-*S. aureus* potential was assessed (Lee et al.). Furthermore, phage Sb-1 (family *Herelleviridae*) was examined as a control agent against rifampin resistant *S. aureus* biofilm upon simultaneous or staggered addition of various relevant antibiotics (Wang et al.). The combination of Sb-1 phage and daptomycin was shown as the most promising in the context of exploitation of the phenomenon called “phage-chemical agent” or “phage-antibiotic” synergy. Related to the abovementioned foulbrood disease control, Brady et al. using state-of-the-art methods demonstrated that phage PL.Ph-1 does not adsorb only to vegetative cells of *P. larvae*, but also to bacterial endospores, which has significant applicative implications.

## Phage Cocktail and Endolysin Design

Bacteriophages show group specificity to bacteria and one phage strain usually cannot infect all strains of one bacterial species. This issue can be overcome by preparation of phage cocktails that contain various phages infecting one species, with different lytic spectra. Haines et al. used ESBL-producing bacteria that cause urinary tract infections as a model to develop a useful method for phage selection and cocktail design, including Direct Spot Test, the Efficiency of Plating assay, the Planktoning Killing assay and the Biofilm assay. Examining 21 myovirus and one siphovirus, Nale et al. designed an optimal cocktail containing three carefully selected phages to combat various swine and poultry *Salmonella* serotypes. The cocktail potency was confirmed both *in vitro* and *in vivo*, using a co-infection and remedial regimen method on *Galleria mellonella* larva model. The authors confirmed the efficacy of the cocktail for treatment and prevention against *Salmonella* infection *in vivo*. Son et al. designed a chimeric endolysin Lys109 efficient against *Staphylococcus aureus*.

## Perspectives and Opinions

Cazares et al. presented an interesting opinion that phage ecology and evolution are usually neglected in phage therapy, particularly bacteria-phage interactions. They pointed out that quorum sensing (QS) can affect bacterial susceptibility to phages, while phage can modulate bacterial cooperation. Accordingly, the recommendation is to carefully examine these interactions to better exploit phage antibacterial properties.

As indicated, Khalid et al. pointed out potential of PT application in developing countries, and similarly was observed by Fauconnier et al. whose opinion is that PT use has been little explored for low-income and middle-income countries. The authors clearly declared that the World Health Organization (WHO) should have a prominent role in the deployment of phage therapy. For instance, the WHO could help promote the knowledge of PT and build a regulatory system for phage products through its vaccines prequalification (PQ) program.

Finally, Pirnay predicted PT destiny in the next 25 years, indicating the possibility for personalized therapies. The author described chain of events that can result in a cell free synthetic phage production—from community efforts, through support of health organizations to the implementation involving Artificial Intelligence and a Distributed Ledger Technology. Although it seems feasible in theory, Pirnay is aware that this is an ideal scenario which can be easily disturbed by many obstacles.

## Author Contributions

All authors listed have made a substantial, direct and intellectual contribution to the work, and approved it for publication.

## Conflict of Interest

The authors declare that the research was conducted in the absence of any commercial or financial relationships that could be construed as a potential conflict of interest.

